# Sufrimiento existencial y autonomía: Aportes a los debates sobre la eutanasia y el suicidio medicamente asistido en Argentina

**DOI:** 10.18294/sc.2026.5970

**Published:** 2026-03-09

**Authors:** Darío Iván Radosta, Matías Paschkes Ronis

**Affiliations:** 1 Doctor en Antropología Social. Becario posdoctoral, Consejo Nacional de Investigaciones Científicas y Técnicas. Investigador, docente, Escuela Interdisciplinaria de Altos Estudios Sociales, Universidad Nacional de San Martín, Buenos Aires, Argentina. diradosta@gmail.com Universidad Nacional de San Martín Escuela Interdisciplinaria de Altos Estudios Sociales Universidad Nacional de San Martín Buenos Aires Argentina diradosta@gmail.com; 2 Doctor en Antropología Social. Docente, Facultad de Ciencias Sociales, Universidad de Buenos Aires. Investigador asociado, Escuela Interdisciplinaria de Altos Estudios Sociales, Universidad Nacional de San Martín, Buenos Aires, Argentina. matiasronis85@gmail.com Universidad Nacional de San Martín Universidad Nacional de San Martín Escuela Interdisciplinaria de Altos Estudios Sociales Buenos Aires Argentina matiasronis85@gmail.com

**Keywords:** Eutanasia, Suicidio Médicamente Asistido, Autonomía Personal, Actos Normativos, Argentina, Euthanasia, Medically Assisted Suicide, Personal Autonomy, Normative Acts, Argentina

## Abstract

El presente artículo nace de la necesidad de reflexionar críticamente sobre la experiencia subjetiva de quien solicita la eutanasia y las tensiones éticas entre la necesidad de claridad conceptual que requiere una normativa jurídica y lo específico e incuantificable de la experiencia humana vinculada al sufrimiento y la fragilidad. En este sentido, este ensayo busca aportar a los debates sobre la temática a partir de las herramientas conceptuales que brinda la filosofía moral, particularmente en lo que respecta a los análisis de Charles Taylor sobre la construcción del self y lo propuesto por Paul Ricoeur con relación a la noción de autonomía. Tomando como punto de partida los anteproyectos de ley para la regulación de la eutanasia y el suicidio médicamente asistido presentados en Argentina entre los años 2021 y 2024, recuperamos los aportes de los autores mencionados para reflexionar críticamente acerca de dos puntos que consideramos de relevancia ética y jurídica para este tipo de propuestas: 1) el sufrimiento existencial como causal de solicitud de eutanasia y 2) el cruce entre autonomía y fragilidad como horizontes del sujeto de derecho.

## Abstract (en)

This article arises from the need to critically reflect on the subjective experience of those who request euthanasia, as well as on the ethical tensions between the conceptual clarity required by legal regulation and the specific, unquantifiable dimensions of human experience linked to suffering and fragility. In this sense, this essay seeks to contribute to current debates on the topic through the conceptual tools offered by moral philosophy, particularly Charles Taylor’s analyses of the construction of the self and Paul Ricoeur’s reflections on the notion of autonomy. Taking as a point of departure the draft bills presented in Argentina between 2021 and 2024 to regulate euthanasia and medically assisted suicide, we draw on the contributions of these authors to critically examine two issues we consider ethically and legally significant for such proposals: (1) existential suffering as grounds for requesting euthanasia, and (2) the intersection between autonomy and fragility as defining horizons of the subject of rights.

## Introducción

El primer cuarto del siglo XXI se ha visto atravesado por un aumento sostenido de los países que han creado marcos regulatorios que institucionalizan jurídicamente la toma de decisiones en el final de la vida vinculadas con la eutanasia y el suicidio médicamente asistido. Países Bajos y Bélgica, en 2002; Luxemburgo, en 2009; Canadá, en 2016; Alemania, en 2020; España y Nueva Zelanda, en 2021; Austria, en 2022; Portugal, en 2023; varios estados de Australia, entre 2021 y 2023; y varios estados de EEUU, entre 2008 y 2021, han llevado adelante debates legislativos que, al menos en los casos mencionados, concluyeron con la aprobación de una ley de eutanasia o suicidio médicamente asistido (o al menos su despenalización). 

Latinoamérica no se encuentra exenta de estas discusiones. Colombia tuvo una temprana despenalización de la eutanasia en 1997, a la que le ha seguido, recientemente, su despenalización en Ecuador en 2024 y la elaboración de un proyecto de legalización de la eutanasia que ha sido aprobado en Uruguay en 2025. Puntualmente este último hecho ha reavivado en Argentina un debate que se viene sosteniendo hace aproximadamente cuatro años, cuando en 2021 los diputados Cornejo, Latorre y Cacace, entre otros, presentaron el anteproyecto de *Ley de buena muerte: Regulación de la eutanasia*^1^. A este anteproyecto le siguieron el presentado ese mismo año por las diputadas Estévez, Brawer y Carrizo junto a otros legisladores, llamado “Ley Alfonso”, *Derecho a la prestación de ayuda para morir dignamente*^2^, y el presentado en 2022 por el diputado Cobos, titulado *Regulación de la eutanasia y la muerte asistida*^3^. Ese mismo año, Brawer y otros presentaron un nuevo proyecto para legalizar la eutanasia y el suicidio asistido^4^, con las posteriores presentaciones de Latorre y otros en 2023^5^ y Pichetto en 2024^6^. Si bien en 2025 se han presentado anteproyectos para tratar el tema, debido a la pérdida de estado parlamentario de los ya mencionados, estos fueron elaborados posteriormente al desarrollo y escritura de este artículo.

Como se puede ver, el periodo comprendido entre los años 2021 y 2024, Argentina ha contado con la presentación de múltiples anteproyectos que buscan legislar en materia de eutanasia y/o suicidio médicamente asistido, siendo este un debate que, aunque traccionado desde diferentes fuerzas políticas de manera independiente entre sí, aún no ha sido saldado en el terreno jurídico. 

Aunque ninguno de estos anteproyectos haya llegado todavía al momento de su tratamiento legislativo, existen diferentes análisis éticos y jurídicos que han tratado de abordar específicamente la cuestión de la eutanasia y el suicidio médicamente asistido en Argentina y América Latina. Entre los puntos fundamentales se presentan los conflictos médicos, éticos y legales en torno a la noción de autonomía y sus implicancias para la legalización de la eutanasia en Argentina^7^^,^^8^^,^^9^; las convergencias entre los modelos legislativos respecto de la despenalización de la eutanasia en Colombia, Ecuador y Perú, con la búsqueda por pensar en un posible modelo latinoamericano de asistencia en el morir^10^; y el abordaje de las actitudes y pensamientos en torno a la eutanasia en profesionales de la salud en México^11^. Otros trabajos han buscado analizar las bases éticas de la eutanasia, tomando como referencia los fundamentos del principalismo, particularmente los referidos al respeto por la autonomía y la no-maleficencia^12^.

Si bien estos trabajos permiten profundizar conceptualmente el debate ético y jurídico en torno a la posibilidad de legislar sobre la eutanasia y el suicidio médicamente asistido en Argentina, consideramos que hay un punto de reflexión^13^ sobre el cual es necesario insistir, dado que es escasamente abordado en los anteproyectos presentados hasta el momento en el país. Nos referimos particularmente a las consideraciones sobre la experiencia subjetiva de la persona que es objeto de estas normativas, quien solicita la eutanasia. Creemos que es pertinente ahondar sobre este aspecto de la toma de decisiones en el final de la vida, de modo de reflexionar críticamente sobre las posibilidades de legislar en materia de eutanasia y suicidio médicamente asistido, al mantener una tensión constante entre la necesidad de claridad conceptual que requiere una normativa jurídica y lo específico e incuantificable de la experiencia humana vinculada al sufrimiento y la fragilidad.

En este sentido, el objetivo de este artículo es, tomando como punto de partida los anteproyectos de legalización de la eutanasia y el suicidio médicamente asistido presentados en Argentina, reflexionar críticamente acerca del lugar de la experiencia subjetiva de la persona en este tipo de marcos jurídicos, haciendo énfasis en dos puntos fundamentales: la noción de sufrimiento existencial como causal de solicitud de eutanasia y la convergencia conceptual entre las ideas de autonomía y fragilidad, y sus implicancias en este tipo de legislaciones. Para llevar adelante esta reflexión nos serviremos principal, pero no únicamente, de las herramientas conceptuales provenientes de la filosofía moral, particularmente, los análisis sobre la construcción del *self* en la obra de Charles Taylor^14^ y los escritos sobe autonomía y fragilidad pertenecientes a Paul Ricoeur^15^. Intentaremos brindar también, en lo posible, herramientas que permitan tender puentes entre la reflexión crítica sobre el sufrimiento y la fragilidad humanas y el requerimiento de precisión conceptual que conlleva el desarrollo de una normativa jurídica. Esto lo haremos, igualmente, sin suponer que la experiencia subjetiva de lo humano puede ser reducida a categorías absolutizantes o estáticas y priorizando siempre mantener la tensión entre esos dos registros (el jurídico y el experiencial) antes que resolviéndola. 

## Eutanasia y sufrimiento existencial

Cuando se analizan cuáles son los requisitos para solicitar la eutanasia o el suicidio médicamente asistido en los anteproyectos de ley que se han formulado en Argentina (y generalmente en los marcos normativos aprobados en otros países), casi siempre se parte de la necesidad de que la persona en cuestión esté atravesando una enfermedad grave e incurable, o bien un padecimiento grave, crónico e imposibilitante. Si bien varían ligeramente entre sí, la definición general de estas situaciones refiere, para el caso de las enfermedades graves, a “alteraciones irreversibles de la salud […], certificadas por un médico, con pronóstico fatal en un plazo breve y que no sean susceptibles de tratamientos curativos”^3^ mientras que los padecimientos son entendidos como “limitaciones de la autonomía física y la capacidad de expresión y relación, que llevan asociados un sufrimiento psíquico o físico constante e intolerable, sin posibilidad de curación o mejoría apreciable”^1^^,^^2^. 

Como puede verse, la definición misma de una enfermedad grave incluye la necesidad de certificación de un médico responsable, por lo que, aunque hubiere discusiones posibles dentro del mismo sistema médico acerca de los pronósticos o plazos en que una enfermedad específica puede terminar con la vida de una persona, habría en cierto sentido mecanismos que permiten estipular con precisión relativa cuál es el grado de sufrimiento y posibilidad de cura frente a ciertos diagnósticos. Si bien no es este el punto sobre el cual nos queremos concentrar en este apartado, como hemos señalado, esto no significa que no existan tensiones en torno a cómo se definen los criterios de “terminalidad” de una enfermedad o cómo se evalúa el umbral de sufrimiento necesario para efectivamente poder solicitar la eutanasia: ¿frente al diagnóstico?, ¿cuándo quedan aproximadamente dos años de vida?, ¿un año?, ¿seis meses?, ¿frente a un síntoma específico que la persona no tolere?, ¿la definición de esa tolerancia es subjetiva o es una evaluación médica?

Quisiéramos centrarnos, entonces, en la definición, quizá más problemática e imprecisa (por su carácter eminentemente subjetivo), de padecimiento grave, crónico e imposibilitante. Tomemos como ejemplo la limitación de la autonomía física, presente en los tres anteproyectos. Una persona puede pasar su vida entera sin tener la capacidad de caminar por sus propios medios, mientras que otra, quizá dedicada toda su vida a correr (como puede ser quizá un maratonista o deportista), puede padecer un sufrimiento psíquico intenso frente a la idea de perder el movimiento en sus piernas. ¿Cuál es la diferencia fundamental entre ambos casos? La evaluación subjetiva que se hace de la situación, en términos de sufrimiento, en función del significado y el sentido que tiene para cada sujeto ese evento. Y es aquí donde las herramientas conceptuales provenientes de filosofía moral pueden arrojar luz sobre estas cuestiones, para poder tensionar los términos jurídicos que terminan componiendo los marcos legales para la toma de decisiones. Todo padecimiento humano, entonces, es relativo al sentido, y es allí donde la noción de *sufrimiento existencial* puede ser útil para pensar la eutanasia y el suicidio médicamente asistido.

En principio tomaremos algunas ideas provenientes del filósofo Charles Taylor, de forma de definir con mayor precisión conceptual a qué nos referimos con la idea del humano como sujeto de sentido. En principio, cabe destacar que, al intentar definir las particularidades de la persona humana, Taylor marca como punto crucial nuestra necesidad de asignar un propósito a las cosas, lo que genera, en cierto sentido, que las cosas “nos importen” en diferente medida o tengan un significado con relación a nuestra posición en el mundo^14^. La persona humana es, ante todo, un agente moral, cuya agencia -su capacidad de actuar en el mundo- requiere de una conciencia reflexiva acerca de los estándares por lo que uno vive y que generalmente se definen, aclara Taylor, por una predisposición de los seres humanos de orientarse moralmente, sea acercándose a un bien o alejándose de un mal. El sentido del propio *self*, concluye parcialmente el autor, es el sentido de dónde uno se encuentra con relación a esos estándares de vida, cuyo carácter moral se proyecta constantemente desde nuestras conciencias hacia el mundo (lo cual explicaría, en parte, el porqué de una significancia diferencial de los eventos entre diferentes personas, dado que han forjado agencias morales disímiles a lo largo de su trayectoria de vida).

Estas ideas, que pueden revestir de cierta complejidad conceptual, tienen el potencial de ayudarnos a comprender algunas de las particularidades de la toma de decisiones en el final de la vida, particularmente en torno a la necesidad de definir un padecimiento grave, incurable e imposibilitante. 

El ser humano, según los análisis de Taylor, es un ser de sentido; un ser para el cual otros seres, los eventos y las cosas, sea como sea que los defina moralmente, tienen un significado en función de si constituyen o no una parte relevante de su trayectoria de vida. ¿Qué sucede, entonces, cuando un ser humano, un ser ligado existencialmente al sentido, comienza a perder, poco a poco o bruscamente, las fuentes de esa significancia? Para responder esta pregunta tomemos como ejemplo una escena de la última película del director Pedro Almodóvar “La habitación de al lado”: una de las mayores preocupaciones de la protagonista, Martha, quien padece un cáncer que no ha respondido al tratamiento, es la pérdida gradual de los placeres que antes le daban significado a su vida. No poder escribir o leer, o incluso escuchar música, sin pensar en su enfermedad, se le presenta como un escenario sumamente angustiante. ¿Forma parte esto de un padecimiento grave, incurable e imposibilitante?, ¿es este un sufrimiento psíquico o físico tal que uno pueda solicitar asistencia para terminar con su vida?, ¿cómo conceptualizar jurídicamente esta angustia de un modo que nos permita evaluar la toma de decisiones en el final de la vida?

Definitivamente no somos los primeros en sostener una propuesta de este calibre, teniendo en cuenta la complejidad de la categoría de sentido en la existencia humana. Dentro de la bibliografía especializada en el cuidado en el final de la vida, es común encontrar categorías como *sufrimiento existencial* o *sufrimiento espiritual* (*existencial and spiritual suffering or distress*) para definir esa “profunda sensación de pérdida, desesperanza e impotencia que puede surgir al enfrentarse a la muerte”^16^, la “ausencia de significado, propósito, esperanza y conexión social, y amenaza la identidad personal”^17^, una “sensación desolada de falta de sentido”^18^, un “dolor relacional derivado de pérdidas existenciales”^19^, o “un dolor causado por la extinción del ser y del sentido del yo”^20^. Bates^21^, por otra parte, en una indagación de este concepto, marca que los aspectos más comunes de la definición de *sufrimiento existencial* se vinculan con la falta de significado o propósito, la pérdida de conexión con otros, la crisis de identidad o del ser y la pérdida de esperanza, autonomía o sentido del tiempo. Incluso desde la Sociedad Española de Cuidados Paliativos (SECPAL), en un libro dedicado a la noción de espiritualidad en clínica, se define este tipo de sufrimiento como asociado a una “desintegración del *self*”^22^. Si se toma la definición de *self* que se deriva de los análisis de Taylor, es comprensible y coherente que ciertos fenómenos asociados al final de la vida se nos presenten como fuentes de sufrimiento existencial y que nos provoquen, de esta manera, un daño psíquico que podría considerarse subjetivamente como intolerable. 

Incluso, autores como Savulescu^23^ han elaborado propuestas que buscan aproximarse a la toma de decisiones en el final de la vida a partir de este tipo de conceptualizaciones, proponiendo que el “sufrimiento existencial, el envejecimiento y la pérdida de ideales propios”^23^^)^ pueden ser entendidas como buenas razones para desear terminar con la propia vida, o sea, razones válidas para solicitar una eutanasia o suicidio médicamente asistido.

El objetivo de estas reflexiones es, como hemos señalado, recuperar parte de la complejidad de lo que implica el sufrimiento humano, dada su intrínseca indeterminación en categorías estáticas. Uno podría pensar que la noción de “sufrimiento psíquico” presente en las formulaciones de los anteproyectos podría ser un paraguas lo suficientemente extenso como para contener este tipo de situaciones, pero efectivamente el carácter normativo de una ley, en ocasiones, deja por fuera de consideración la experiencia subjetiva de las personas a quienes está dirigida. Recuperar esa complejidad del sufrimiento humano requiere, por ende, sostener esa tensión entre lo inabarcable de la experiencia, propio de la realidad, y la necesidad jurídica de trabajar con categorías asimilables. 

Nuestra propuesta, sin embargo, también busca tratar de tender puentes entre estos dos registros -el jurídico y el filosófico-, con el fin de dar lugar a propuestas que superen los anteproyectos hasta el momento presentados en el país. Si bien el sufrimiento humano es un fenómeno situado, relacional y no reducible a categorías estáticas o indicadores cuantitativos, existen herramientas que, utilizadas con la precaución que requieren, podrían servir como puentes entre el lenguaje médico, el jurídico y el filosófico. A este respecto, instrumentos como “*The meaning in life questionnaire*”^24^, la “*Existencial distress scale* (EDS)” basada en los trabajos de Lo, *et al*.^25^, el “*Patient dignity inventory*”^26^ o la “*Demoralization scale* (DS)”^27^, entre otros, se han elaborado con el fin de intentar evaluar el impacto subjetivo sobre el *self* que tienen los diferentes componentes prototípicos de una situación de final de vida.

No estamos queriendo decir con esto que aplicar irreflexivamente un instrumento de evaluación sea la solución para comprender las complejidades de la toma de decisiones en el final de la vida, ni que el camino sea el de cuantificar el sufrimiento humano en escalas o indicadores o que esto vuelva el sufrimiento más “verdadero” o “legible”. Pero consideramos que, utilizados desde una óptica que sostenga la tensión sobe la cual venimos hablando, estas herramientas podrían ser una base desde la cual pensar la manera de traducir la experiencia humana -inagotable en su contenido- a lenguajes que puedan formar parte de la normativa jurídica a partir de la cual se construya un marco para legislar sobre la eutanasia y el suicidio médicamente asistido en Argentina.

## Eutanasia, autonomía y fragilidad

Lo desarrollado en el apartado anterior a partir de los análisis de Taylor nos permite, en principio, profundizar el debate sobre la cuestión de la dignidad y la autonomía -en tanto conceptos nodales en las controversias sociales y legales acerca de la eutanasia- proponiendo una noción de persona humana en tanto ser existencialmente ligado al sentido. Esta noción, como mostramos, permite en principio repensar los criterios presentados en los anteproyectos de ley en Argentina, fuertemente centrados en una mirada médica que garantice la existencia de una enfermedad grave e incurable. 

Por otro lado, dicha noción permite salir de las encrucijadas que plantea una noción de autonomía y dignidad fuertemente individualistas presentes -aunque no reconocidas como tales- tanto en quienes defienden estos proyectos como en quienes los objetan. Centrémonos aquí en estos últimos. Vamos a tomar como referencia, en especial, el artículo del Dr. Jorge Nicolás Lafferriere, director del Centro de Bioética, Persona y Familia, que analiza críticamente los tres anteproyectos. Según este autor:

“La eutanasia o el suicidio asistido legalizado no son la solución a sus sufrimientos e implican un quiebre del principio de la inviolabilidad del derecho a la vida y la instauración de un radical individualismo que se vuelve indiferente ante el que sufre y se limita a ofrecer el camino del descarte”.^28^

El individualismo o utilitarismo, que este autor denuncia, supone que el ejercicio de la eutanasia prioriza “el derecho a la muerte” -que, según su perspectiva, no existe como tal- por sobre los cuidados y, en ese sentido, pone en peligro la convivencia social. Para Lafferriere

“En los proyectos de ley subyace una concepción de dignidad asociada a la ‘calidad de vida’ y subordinada a su utilidad y que llevaría a clasificar a las personas entre las que son dignas y las que no son dignas de vivir”.^28^

Su argumento es más que interesante porque nuevamente centra las críticas en un concepto de dignidad ligado a la autonomía y la calidad de vida, mientras que para él la dignidad es inherente a toda persona y no se puede reducir a la autonomía. Más aún, Lafferriere resalta que el legislador muchas veces debe limitar la autonomía humana para proteger la dignidad^28^. 

Esta perspectiva de la dignidad se encuentra asociada a la fe católica de “*Dignitas infinita*” y a una concepción de la dignidad ontológica según la cual le corresponde “a la persona como tal por el mero hecho de existir y haber sido querida, creada y amada por Dios”^29^. Ahora bien, resulta importante resaltar que la Doctrina de la Fe -tal como se presenta en la Declaración “*Dignitas infinitas*”^29^- realiza una cuádruple distinción en el concepto de dignidad, agregando a la dignidad ontológica, la dignidad moral, ligada al ejercicio de la libertad; la dignidad social, ligada a las condiciones concretas de vida; y la dignidad existencial, vinculada a la experiencia subjetiva de la propia condición de vida. Estas tres últimas distinciones resultan sumamente valiosas a la hora de pensar: 1) la articulación entre la dignidad y autonomía; 2) las condiciones sociales concretas en las que la vida se desarrolla; 3) la importancia del sufrimiento existencial, tal como lo trabajamos en el apartado anterior. Sin embargo, estas tres últimas distinciones resultan luego reducidas en importancia frente a la dimensión “ontológica” que recae en un punto de vista individualista y abstracto, aunque Lafferriere la presente como un criterio ético y jurídico objetivo “para poder discernir las exigencias de justicia para las situaciones y casos que enfrenta un paciente en el final de su vida”^28^. El concepto individualista supone, en este caso, que dicho concepto ontológico de dignidad se vincula con un concepto de persona como “sustancia individual de naturaleza racional”^29^. Ahora bien, resulta llamativo acá que la sustancia individual refiera a un sujeto que “habiendo recibido la existencia de Dios, ‘subsiste’, es decir, ejerce la existencia autónomamente”^29^, volviendo a ligar dignidad y autonomía bajo una noción individualista. 

Frente a estas argumentaciones abstractas e individualistas que obturan cualquier debate, consideramos necesario proponer perspectivas centradas en las condiciones sociales y subjetivas concretas en las cuales la realización de la dignidad y la autonomía son posibles y realizables. Lejos de cualquier esencialismo o dogmatismo, esto supone volver a una reflexión en torno al sujeto de derecho para la cual, como muestra Paul Ricoeur^15^, la autonomía es su condición de posibilidad a la vez que es el horizonte de la tarea jurídica. La cuestión no radica en marcar criterios basados en perspectivas abstractas; sino, por el contrario, en poder generar los dispositivos legales e institucionales que coadyuven a la realización -nunca completa ni total- de la autonomía personal. 

Tal como señala Ricoeur, la autonomía no es la de un ser realizado, sino justamente es la de un ser frágil, vulnerable. Y es, por lo tanto, dicha vulnerabilidad la que hace de la autonomía una tarea de la práctica jurídica. Vulnerabilidad que se expresa, por ejemplo, en la capacidad de actuar -la potencia de obrar- que lejos de ser demostrada como tal, supone siempre una “confianza” que requiere a otro, un otro capaz de “dar confianza”. En ese sentido, cuando debatimos sobre el derecho a la eutanasia debemos centrarnos no solo en las condiciones concretas en las cuales se realiza o concibe la dignidad, sino también en las que la autonomía puede ser asumida por un sujeto frágil al que le toca atravesar una experiencia límite como, en una situación de la cual no es responsable, asumir la decisión de poner fin a su vida. Dicha “decisión” no puede ser dejada simplemente en sus manos en tanto “individuo autónomo y racional”, sino que nos compete a todos en tanto sociedad. Su autonomía debe ser -para ser efectivamente realizada- acompañada por quienes él o ella desee, quienes le brinden confianza. El cuidado no se restringe entonces a los cuidados paliativos -los cuales son, no hace falta aclarar, indispensables garantizar-, sino también al cuidado que sostiene al sujeto en su fragilidad para poder obrar y sostener su identidad, respetando su sufrimiento existencial y sus decisiones en torno a su propia dignidad en ese momento crucial de la vida que constituye el morir. 

En ese sentido, consideramos que no se debe escindir los conceptos de autonomía y dignidad, tal como lo plantea Lafferriere, puesto que la dignidad, lejos de ser un problema filosófico abstracto u ontológico, se hace carne en las decisiones que un sujeto toma acerca de cómo quiere vivir y dirigir su vida de acuerdo con sus valores en situaciones concretas. La idea de que se puede y debe limitar la autonomía para proteger la dignidad supone un legislador que actúe en beneficio de la persona (y defina el beneficio y la dignidad de esa persona) más allá de los intereses y valores. Y esto a la vez presupone dos cuestiones más: 1) que dicho beneficio constituye el objetivo de la autonomía y que cuando no se actúa con base en ese criterio no hay autonomía; y 2) que puede haber un criterio jurídico objetivo para discernir en esos casos. Siguiendo los planteos Dworkin^30^ podemos decir que la autonomía como horizonte y posibilidad no tiene como objetivo promover el beneficio o el interés del individuo -de hecho, muchas decisiones autónomas se toman contra el propio interés-, sino que “el valor de la autonomía radica en el esquema de responsabilidad que crea”^30^. El autor plantea así una concepción de la autonomía basada en la integridad de la persona que no se reduce a su simple interés o beneficio, sino a la capacidad del propio agente de tomar decisiones y dirigir su vida “en el contexto estructurado de la totalidad de una vida organizada alrededor de una concepción coherente del carácter y la convicción”^30^. 

De esta manera, el derecho a la autonomía basada en la integridad y coherencia nos conduce nuevamente a Ricoeur y su noción de identidad narrativa. Para este filósofo:

“…la gestión de la propia vida, como la historia susceptible de coherencia narrativa, representa una competencia de alto nivel que debe ser considerada como un componente fundamental de la autonomía del sujeto de derecho”.^15^

Por lo tanto, un ser autónomo es un sujeto capaz de conducir su vida de acuerdo con la idea de coherencia narrativa. Las experiencias límites, como el sufrimiento existencial o enfermedades amenazantes para la vida, nos permiten visibilizar la fragilidad en lo que hace al sostenimiento de la propia identidad del sujeto. 

Dicho esto, pensar la autonomía como horizonte requiere entonces superar nociones esencialistas e individualistas de la dignidad y de la identidad que terminan por diluir dicho derecho. Supone avanzar en el reconocimiento de la fragilidad de los sujetos para crear así los dispositivos de acompañamiento necesarios para que la autonomía, en tanto horizonte, pueda ejercerse con responsabilidad aún -y en especial- en las situaciones y decisiones más difíciles en la vida de una persona. 

## Reflexiones finales

El recorrido realizado en este artículo buscó situar la discusión sobre la eutanasia y el suicidio médicamente asistido en un cruce de perspectivas donde confluyen los debates normativos, las tensiones bioéticas y las aproximaciones conceptuales propias de la filosofía moral. La intención no ha sido ofrecer respuestas cerradas ni fórmulas de aplicación inmediata, sino contribuir a precisar categorías, visibilizar dilemas y enriquecer el debate en un terreno en el que la experiencia humana se entrelaza inevitablemente con cuestiones filosóficas, jurídicas, médicas y culturales.

Uno de los aportes centrales que hemos querido subrayar es la necesidad de dar un lugar explícito al sufrimiento existencial dentro de las discusiones normativas. Si bien los marcos legislativos suelen apoyarse en categorías médicas como la enfermedad grave y/o incurable, esta mirada resulta insuficiente para dar cuenta de la complejidad de la experiencia subjetiva de quienes atraviesan el final de la vida. La condición humana se encuentra inevitablemente ligada al sentido y, cuando este se erosiona o se pierde, se genera un tipo de padecimiento que no puede ser reducido a parámetros clínicos. Incorporar el sufrimiento existencial como categoría jurídica no significa relativizar la norma ni volverla excesivamente ambigua, sino dotarla de herramientas conceptuales más finas que permitan reconocer la diversidad de trayectorias vitales y de modos de experimentar nuestra fragilidad.

En paralelo, hemos mostrado que la discusión sobre la autonomía, lejos de agotarse en un enfoque liberal-individualista, requiere ser repensada bajo el horizonte de la vulnerabilidad humana y las relaciones con los otros. Las críticas que reducen la eutanasia a una expresión de individualismo extremo suelen desconocer que las decisiones sobre el morir están siempre situadas en un entramado de vínculos, cuidados y apoyos. La autonomía no se ejerce en el vacío: es una práctica que necesita ser acompañada, sostenida y reconocida. Retomar aquí a autores como Ricoeur o Dworkin permite entender que la autonomía es, a la vez, condición de posibilidad y horizonte, y que se despliega en diálogo con la fragilidad y la necesidad de cuidado.

De este modo, dignidad y autonomía no deben pensarse como conceptos separados, sino como nociones entrelazadas que adquieren sentido en contextos sociales concretos. La dignidad no se agota en una definición ontológica abstracta ni puede imponerse desde una visión trascendente que desconozca la voz de los sujetos; se hace carne en las decisiones vitales, en la coherencia narrativa que cada persona busca mantener hasta el final, incluso en medio de la enfermedad, el dolor o la pérdida de capacidades.

El caso argentino, con sus avances parciales en derechos de los pacientes y cuidados paliativos, y sus anteproyectos de ley aún sin tratamiento, muestra que el debate está abierto y que las ciencias humanas tienen un rol fundamental que cumplir: aportar marcos conceptuales que iluminen los dilemas y ayuden a construir normativas más sensibles a la experiencia humana. Al mismo tiempo, los desarrollos internacionales y las producciones culturales recientes nos recuerdan que se trata de una discusión global, que interpela simultáneamente a los sistemas legales, a las comunidades de cuidado y a la sociedad en su conjunto.

En definitiva, pensar la eutanasia y el suicidio médicamente asistido desde las ciencias humanas implica no eludir las tensiones, sino habitarlas críticamente. Se trata de reconocer que el morir no es solo un hecho biológico o médico, sino un acontecimiento social y cultural, en el que se juegan concepciones de autonomía, dignidad, vulnerabilidad y sentido. Solo a partir de este reconocimiento podremos construir marcos normativos y prácticas sociales que acompañen a las personas en los momentos finales de su vida, respetando sus decisiones y sosteniendo, a la vez, la responsabilidad colectiva frente a la fragilidad humana.

## References

[1] Cornejo A, Latorre J, Cacace A (2021). Proyecto de ley: Ley de buena muerte: regulación de la eutanasia (Expte. 4597-D-2021).

[2] Estevez G, Brawer M, Carrizo C, Gaillard C, Macha M, Moreau C, Lampreabe F, López J (2021). Proyecto de ley: “Ley Alfonso”, Derecho a la prestación de ayuda para morir dignamente (Expte. 4734-D-2021).

[3] Cobos JC (2022). Proyecto de ley: Regulación de la eutanasia y la muerte asistida (Expte. 3956-D-2022).

[4] Brawer M, Gollán D, Carro P, Yasky H, Ormachea C, Gaillard C, Landriscini SG, Martínez MR, Hagman I, Bormioli L (2022). Proyecto de ley: Ley de muerte voluntaria médicamente asistida (Expte. 4855-D-2022).

[5] Latorre J, Nieri L, Verasay P, Stolbizer M, García X, Machado L, Banfi K, Finochiaro A, Quetglas F, Barletta M (2023). Proyecto de ley: Buena muerte regulación de la eutanasia (Expte. 1473-D-2023).

[6] Pichetto MA (2024). Proyecto de ley: Régimen legal de asistencia para terminar con la propia vida (Expte. 1473-D-2023).

[7] Graz A, Coso D (2023). Autonomy and the End of Life: Reflections from Medicine and the Law. SAP Health and Policy.

[8] Aranalde G (2025). Medical, ethical, and legal conflicts surrounding euthanasia in Argentina. Its global implications. Public Health Open Access.

[9] Leani L (2025). Proyectos de regulación de la muerte médicamente asistida en Argentina: dignidad, autonomía, discapacidad y el caso de pacientes no terminales. Revista de Bioética y Derecho.

[10] Espericueta L (2025). Analysis of the legal situation regarding euthanasia in Ecuador, Colombia, and Peru: Towards a Latin American model of medical assistance in dying?. Developing World Bioethics.

[11] Álvarez del Rio A, Marván M (2011). On euthanasia: exploring psychological meaning and attitudes in a sample of mexican physicians and medical students. Developing World Bioethics.

[12] Rivera-López E (2017). Is medically assisted death a special obligation?. Journal of Medical Ethics.

[13] Radosta D (2025). Cuerpo y autonomía: Debates sobre la eutanasia en América Latina. Revista Nueva Sociedad.

[14] Taylor C (1999). The concept of a person. In: Human agency and language. Philosophical papers 1.

[15] Ricoeur P (2008). Lo justo 2: Estudios, lecturas y ejercicios de ética aplicada.

[16] Coyle N, Ferrell B (2016). Fast facts for the hospice nurse: A concise guide to end-of-life care.

[17] Bolton L, Seymour J, Gardiner C (2022). Existential suffering in the day to day lives of those living with palliative care needs arising from chronic obstructive pulmonary disease (COPD): A systematic integrative literature review. Palliative Medicine.

[18] Saunders C (1988). Spiritual pain. Journal of Palliative Care.

[19] McGrath P (2003). Creating a language for ‘spiritual pain’ through research: A beginning. Supportive Care in Cancer.

[20] Murata H (2003). Spiritual pain and its care in patients with terminal cancer: Construction of a conceptual framework by philosophical approach. Palliative & Supportive Care.

[21] Bates AT (2016). Addressing existential suffering. BC Medical Journal.

[22] Benito E, Barbero J, Dones M (2015). Espiritualidad en clínica: una propuesta de evaluación y acompañamiento espiritual en cuidados paliativos.

[23] Savulescu J (2015). A simple solution to the puzzles of end of life? Voluntary palliated starvation. Journal of Medical Ethics.

[24] Steger MF, Frazier P, Oishi S, Kaler M (2006). The Meaning in Life Questionnaire: Assessing the presence of and search for meaning in life. Journal of Counseling Psychology.

[25] Lo C, Zimmermann C, Rydall A, Walsh A, Jones JM, Moore MJ, Shepherd FA, Gagliese L, Rodin G (2010). Longitudinal study of depressive symptoms in patients with metastatic gastrointestinal and lung cancer. Journal of Clinical Oncology.

[26] Chochinov HM, Hassard T, McClement S, Hack T, Kristjanson LJ, Harlos M, Sinclair S, Murray A (2008). The patient dignity inventory: A novel way of measuring dignity-related distress in palliative care. Journal of Pain and Symptom Management.

[27] Kissane DW, Wein S, Love A, Lee XQ, Kee PL, Clarke DM (2004). The Demoralization Scale: A report of its development and preliminary validation. Journal of Palliative Care.

[28] Lafferriere JN (2022). Análisis de los proyectos de legalización de la eutanasia y el suicidio asistido en Argentina.

[29] Fernández VM, Matteo A (2024). Declaración del Dicasterio para la Doctrina de la Fe “Dignitas infinita sobre la dignidad humana”.

[30] Dworkin R (1997). La autonomía y el yo demente. Análisis Filosófico.

